# An imaging mass cytometry immunophenotyping panel for non-human primate tissues

**DOI:** 10.3389/fimmu.2022.915157

**Published:** 2022-07-15

**Authors:** Paula Niewold, Marieke E. Ijsselsteijn, Frank A. W. Verreck, Tom H. M. Ottenhoff, Simone A. Joosten

**Affiliations:** ^1^ Department of Infectious Diseases, Leiden University Medical Centre, Leiden, Netherlands; ^2^ Department of Pathology, Leiden University Medical Centre, Leiden, Netherlands; ^3^ Section of Tuberculosis (TB) Research and Immunology, Department of Parasitology, Biomedical Primate Research Centre, Rijswijk, Netherlands

**Keywords:** imaging mass cytometry (IMC), non-human primate (NHP), immunophenotyping (IP), disease model animal, spatial organization, cynomolgus macaque, rhesus macaque (Macaca mulatta)

## Abstract

It has recently become clear that spatial organization contributes to cellular function and that expanding our knowledge on cellular organization is essential to further our understanding of processes in health and disease. Imaging mass cytometry enables high dimensional imaging of tissue while preserving spatial context and is therefore a suitable tool to unravel spatial relationships between cells. As availability of human tissue collected over the course of disease or infection is limited, preclinical models are a valuable source of such material. Non-human primate models are used for translational research as their anatomy, physiology and immune system closely resemble those of humans due to close evolutionary proximity. Tissue from non-human primate studies is often preserved large archives encompassing a range of conditions and organs. However, knowledge on antibody clones suitable for FFPE tissue of non-human primate origin is very limited. Here, we present an imaging mass cytometry panel development pipeline which enables the selection and incorporation of antibodies for imaging of non-human primate tissue. This has resulted in an 18-marker backbone panel which enables visualization of a broad range of leukocyte subsets in rhesus and cynomolgus macaque tissues. This high-dimensional imaging mass cytometry panel can be used to increase our knowledge of cellular organization within tissues and its effect on outcome of disease.

## Introduction

Historically, image-based technologies for analysis of intact tissues, such as immunohistochemistry and immune fluorescence, have been limited to a small number of parameters. Single cell analyses such as flow and mass cytometry analyze more parameters but require tissue dissociation and as a result outcomes lack spatial context. Recent technological developments enable high dimensional analysis of tissue while maintaining spatial information (1). Such analyses have already demonstrated great added value for understanding biological processes taking place in diseases including cancer and tuberculosis (2, 3). However, obtaining tissue samples from human subjects is challenging and the time of infection or disease initiation is often not known. In preclinical animals models it is possible to perform a controlled infection with appropriate controls and sample a wide range of tissues at selected timepoints post infection. Therefore, application of high dimensional imaging technologies in preclinical animal models, such as non-human primates (NHP), can further our understanding of the function of cellular organization in disease resolution or susceptibility. NHP are commonly used for vaccine efficacy studies and to study pathogens with specific tropisms or disease presentation difficult to mimic in species more distantly related to humans. This includes a range of viral infections [Sars-CoV-2, herpes virus, hepatitis viruses, human immune-deficiency virus (4, 5)], neurological conditions [Parkinson’s disease, Alzheimer’s disease (6)] and bacterial infections [*Mycobacterium tuberculosis* (7)].

Institutions performing research with NHP often have extensive archives of formalin fixed paraffin embedded (FFPE) tissue collected from studies over decades. This method of preservation maintains tissue architecture and allows tissue to be kept for years without specific storage requirements (8). Current use of FFPE tissue is limited as formalin fixation and antigen retrieval requirements can be prohibitive for the detection of antigens. However, current technologies, such as imaging mass cytometry (IMC), can capitalize on tissue archives from previous studies to obtain novel pathophysiological insights without animal burden in line with the replace, reduce and refine (3R) principle (9).

IMC is a novel technology that uses metal isotope-tagged antibodies directed against cellular targets of interest on tissue sections (1). Ablation of the tissue in 1-micron segments is followed by a Time-of-Flight (TOF)-based identification of the metal isotopes present per segment, resulting in a spatial representation of these targets in the tissue. Due to the use of metal isotopes IMC suffers less from spillover issues than fluorescence approaches and 40^+^ markers can be incorporated into a single panel. Furthermore, as the metal isotopes do not suffer from bleaching, it is possible to image additional regions of a section at later stages. Applying this technology to archived FFPE tissue from studies performed in NHP will allow detailed analysis of phenotypes of (immune) cells while maintaining spatial context, yielding novel and valuable information. Performing such work in NHP tissue presents a challenge due to the limited knowledge on suitable clones for FFPE NHP tissue. To enable IMC research on NHP samples we adapted the approach previously published by Ijsselsteijn et al. (10) to create a pipeline to efficiently test antibody clone performance and build an IMC panel for FFPE NHP tissue. Here, we present a backbone panel for the detection of major immunological subsets designed to enable expansion and tailoring to specific research questions addressing the impact of spatial relationships on the outcome of immune interactions.

## Materials and methods

### Tissues

NHP samples were obtained from the Biomedical Primate Research Centre (BPRC), Rijswijk, the Netherlands (courtesy of DVM I. Kondova). BPRC is licensed by the Dutch authority to breed non-human primates and to use them for research in areas of life-threatening and disabling diseases without suitable alternatives. BPRC complies to all relevant legislation with regard to the use of animals in research; the Dutch ‘*Wet op de Dierproeven*’ and the European guideline 2010/63/EU. BPRC is AAALAC accredited since 2012.

Tissue samples from purpose-bred rhesus and cynomolgus macaques in the present study, were retrieved from a biobank of materials that become available occasionally, when an animal happens to be indicated for ketamine-sedation and euthanasia for veterinary and animal welfare reason. Thus, the availability of samples was exploited as it occurred, beyond any legal requirement for prior approval of protocol as there was no pre-existing study plan nor any discomfort afflicted to animals for the sake of a research objective. Samples were randomly collected and neither perfusion or inflation of tissue was performed prior to tissue collection.

FFPE blocks of tonsil and lung of rhesus and cynomolgus macaques were cut into 4 µm sections and transferred to silane-coated glass slides (VWR, Radnor, PA, USA) for further analysis.

### Panel design

Antibody selection was based on the aim to enable detection of main leukocyte subsets (B cells, NK cells, ILC, DC and several T cells and macrophage populations) as well as tissue structure (keratin). Two keratin clones were used to maximize detection of cytokeratin types, including cytokeratin 1 - 8, 10, 13 - 16 and 18-19. Clones with good performance in human tissue and high target-sequence homology were chosen to be tested for cross-reactivity in NHP tissue. Where possible clones that have previously been used for IMC application were selected.

### Immunohistochemistry

The method used has previously been described in (Ijsselsteijn, 2019, Front Immunol). Briefly, sections were deparaffinized and rehydrated using xylene and a decreasing ethanol gradient. Next, endogenous peroxidase was blocked using a 0.3% hydrogen peroxidase/methanol solution (Merck Millipore, Burlington, MA, USA). Antigen retrieval was performed by boiling slides in either pH 9 Tris-EDTA or pH 6 citrate buffers (eBioscience, Thermo Fisher Scientific, Waltham, MA, USA) to compare antibody performance under these conditions. After cooling to room temperature, sections were incubated with Superblock solution (Thermo Fisher Scientific) for 30 minutes to reduce non-specific antibody binding and subsequently incubated with primary antibody overnight at 4°C. The next day, sections underwent multiple PBS washes and were incubated with Poly-horseradish peroxidase solution (Immunologic, Duiven, the Netherlands) for 1 hour at room temperature. Detection of antibody binding was performed using DAB+ chromogen (DAKO, Agilent technologies, Santa Clara, CA, USA) and hematoxylin (Sigma-Aldrich, St Louis, MS, USA) was used for counterstaining.

### Antibody to metal isotope conjugation

The MaxPar antibody labelling kit and protocol were used to conjugate 100 µg of various carrier-free IgG antibody to purified lanthanide metals (Fluidigm, San Francisco, CA, USA). ^89^Y is not commercially available in a conjugation ready format and was kindly provided by the Pathology department at the Leiden University Medical Centre (LUMC) and was conjugated according to the Fluidigm protocol. Finally, the conjugated antibodies were eluted in 50 µL W-buffer (Fluidigm) and 50 µL antibody stabilizer (Candor Bioscience, Wangen im Allgau, Germany) with 0.05% sodium azide and stored at 4°C until use. IHC staining, as described above, was performed again after conjugation to assess whether the conjugation process impacted the specificity of the antibodies.

### Imaging mass cytometry staining

Titrations were performed to determine in which condition (4°C overnight or 5 hours at room temperature) and concentration (1:50 or 1:100) antibodies performed optimally. As abundance of epitopes differs per organ, titrations were performed separately for lung and tonsil tissue. Based on titration results, antibodies were either included in the antibody mix for overnight staining at 4°C or the antibody mix for 5-hour staining at room temperature. Staining for IMC was performed as previously described (10). Briefly, 4 µm sections were deparaffinized and rehydrated after which antigen retrieval was performed at pH 6, as this condition was determined to show the best performance for the majority of the antibodies. Next, sections were blocked and then incubated with the antibody mix for overnight incubation at 4°C. The next day, the sections were washed and subsequently incubated with the antibody mix for 5-hour incubation at room temperature. Following this incubation, sections were washed, incubated with DNA Intercalator (Ir) for 5 minutes at room temperature and washed again. The final wash was performed with demi water and slides were dried and stored until acquisition.

### Imaging mass cytometry acquisition

All data mass cytometry data was acquired on the Hyperion mass cytometry system (Fluidigm) at the Flow cytometry Core Facility at the LUMC. A tuning protocol and 3-element tuning slide were used to autotune the system, as per the Hyperion imaging system user guide (Fluidigm). Areas of interest were identified by light microscopy on consecutive H&E slides. Selected areas of 500 x 500 µm were ablated and acquired at 200 Hz, after which data was exported as MCD files. These files were visualized using the MCD viewer.

## Results

**Figure 1:**
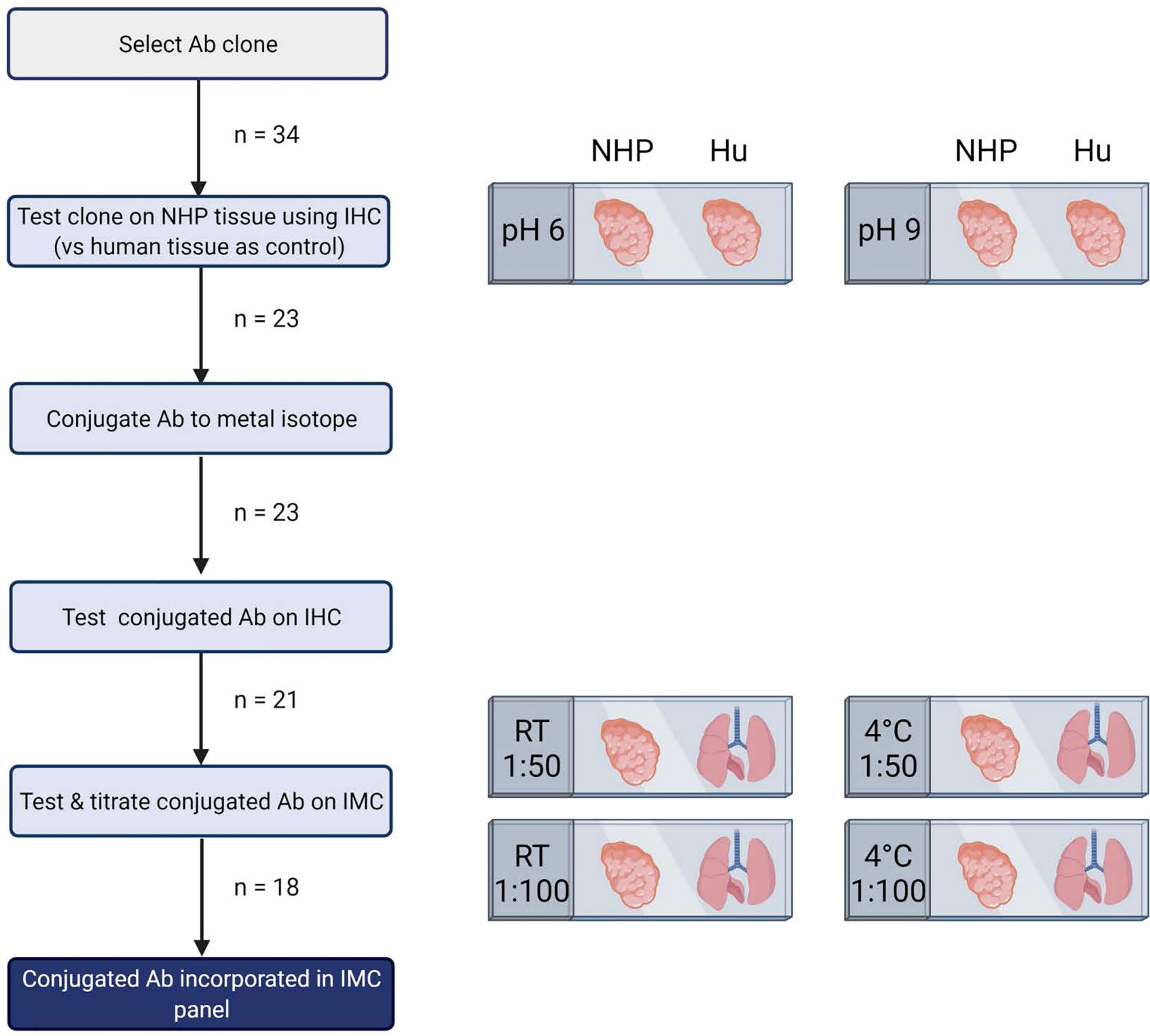
Schematic representation of pipeline for development of imaging mass cytometry panel for NHP tissue. Antibody testing steps and the number of antibodies included in each step are shown. This figure was created with BioRender.com.

**Figure 2:**
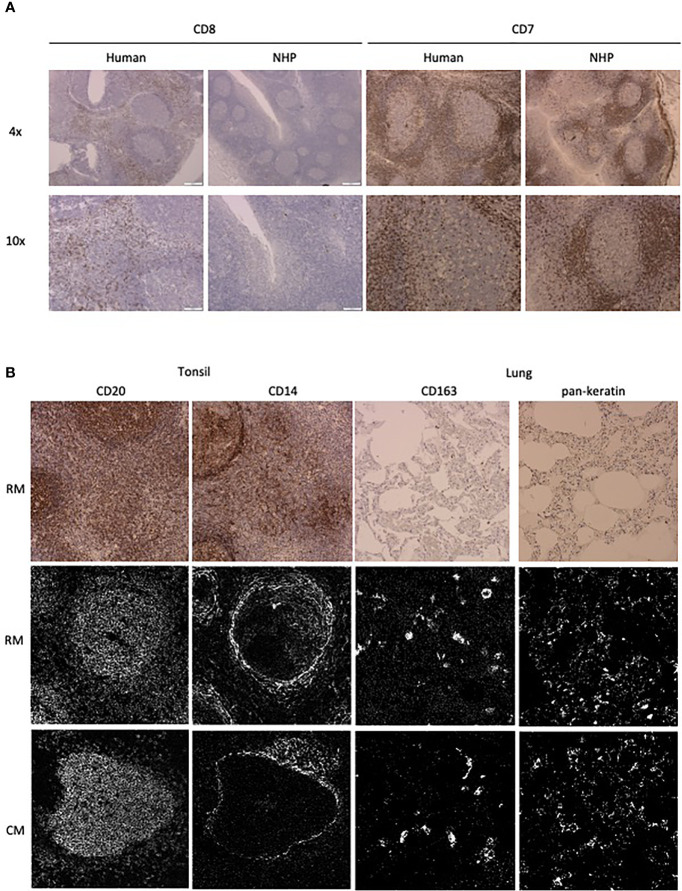
Antibody performance in human and NHP tissue. **(A)** IHC staining pattern of antibody clones in human (left) and NHP (right) tonsil tissue. CD7 and CD8 staining is representative of staining variation between human and NHP tissue. **(B)** Comparison of antibody staining pattern on NHP tissue between IHC and IMC. Tissue relevant markers were chosen, with CD20 and CD14 staining shown in the tonsil and CD163 and pankeratin staining in the lung tissue. IHC staining is shown in rhesus macaque tissue (top row) and IMC staining is shown for both rhesus (middle row) and cynomolgus macaque (bottom row) tissue.

The number of available antibodies known to function in NHP FFPE tissue is limited and more abundantly available antibodies evaluated for other applications such as flow cytometry or IHC on frozen tissue often fail to recognize epitopes in formalin fixed tissue. Therefore, we selected anti-human antibody clones that were previously demonstrated to work on FFPE tissue with high target sequence homology with NHP species to test their cross-reactivity in NHP tissue. We adapted the method employed by Ijsselsteijn et al. (10) to test the target specificity in the tissue of interest by immunohistochemistry (IHC), by staining rhesus macaque tissue sections side-by-side with human tissue as a positive control (**Figure 1**). In total, 34 antibody clones with specificities for 27 different markers were assessed for staining quality in NHP tissue by IHC and 23 of these showed specific staining for the target of interest. Examples are shown in **Figure 2A**; the CD8 clone worked on human FFPE tissue, but was not cross-reactive in NHP tissue, while CD7 staining was shown in tonsil tissue from both species. An alternative clone for CD8 that was cross-reactive in NHP was identified. Excluded antibody clones are listed in **Supplementary Table 1**. The 23 cross-reactive antibodies of interest were conjugated to lanthanide isotopes and their performance in IHC staining was compared to their unconjugated format. Two antibody clones showed no specific signal post-conjugation and were excluded (**Figure 1**). The antibodies were titrated for optimal concentration and staining condition for use on IMC in both lung and tonsil tissue, and staining patterns were compared to IHC to confirm specificity (**Figure 2B**). Three antibodies that performed well on IMC were not incorporated in the current panel but are mentioned here as they are suitable for use on NHP tissue (**Supplementary Table 1**). The final 18-marker panel is shown in **Table 1** and contains structural (cytokeratin), lymphocyte (CD3, CD20, CD7, CD4, CD8, γδTCR), myeloid (CD14, CD68, CD11b, CD206, CD163) and DNA (Intercalator) markers enabling detection of B cells, ILC, DC, monocytes and several subsets of T cells (CD4^+^, CD8^+^, γδTCR^+^) and macrophages (tissue-resident and alveolar). The panel was designed as a general broad phenotyping panel with space to incorporate markers of interest for particular tissues or research questions. Lineage markers have been conjugated to dimmer metal isotopes, thereby leaving space on brighter isotopes for potentially less abundant markers of interest.

**Table 1:** 18-marker panel for imaging mass cytometry, with antibody-metal combination, antibody clone and staining conditions for tonsil and lung tissue.

Target	Clone	Isotope	Tonsil			Lung			Species
			Time	Temp	Dilution	Time	Temp	Dilution	
pan-cytokeratin	AE1/AE3 and C11	89Y	ON	4°C	50	ON	4°C	100	RM, CM
CD45	D9M8I	115In	ON	4°C	100	5h	RT	100	RM, CM
HLADR	TAL-1B5	141Pr	5h	RT	100	5h	RT	100	RM, CM
CD4	EPR6855	142Nd	5h	RT	100	5h	RT	50	RM, CM
CD11b	D6X1N	143Nd	5h	RT	100	5h	RT	100	RM, CM
CD20	H1	144Nd	5h	RT	100	5h	RT	100	RM, CM
CD16	EPR16784	145Nd	5h	RT	100	5h	RT	100	RM, CM
CD8α*	D8A8Y	146Nd	ON	4°C	50	ON	4°C	50	RM, CM
CD123	IL3RA/2947R	147Sm	5h	RT	50	5h	RT	100	RM, CM
γδTCR	H41	148Nd	5h	RT	50	ON	4°C	100	RM, CM
CD3ϵ	D7A6E	156Gd	5h	RT	100	5h	RT	100	RM, CM
CD14	D7A2T	163Dy	ON	4°C	100	ON	4°C	50	RM, CM
Calprotectin	MAC387	171Yb	5h	RT	100	ON	4°C	100	RM, CM
CD206	EPR22489-7	172Yb	5h	RT	100	5h	RT	50	RM, CM
CD7	EPR4242	173Yb	ON	4°C	100	ON	4°C	50	RM, CM
CD68	D4B9C	174Yb	ON	4°C	100	ON	4°C	50	RM, CM
CD163	Edhu-1	175Lu	5h	RT	100	ON	4°C	50	RM, CM
Histone H3	D1H2	209Bi	ON	4°C	50	ON	4°C	50	RM, CM

*Purchased conjugated format from Fluidigm.

**Figure 3:**
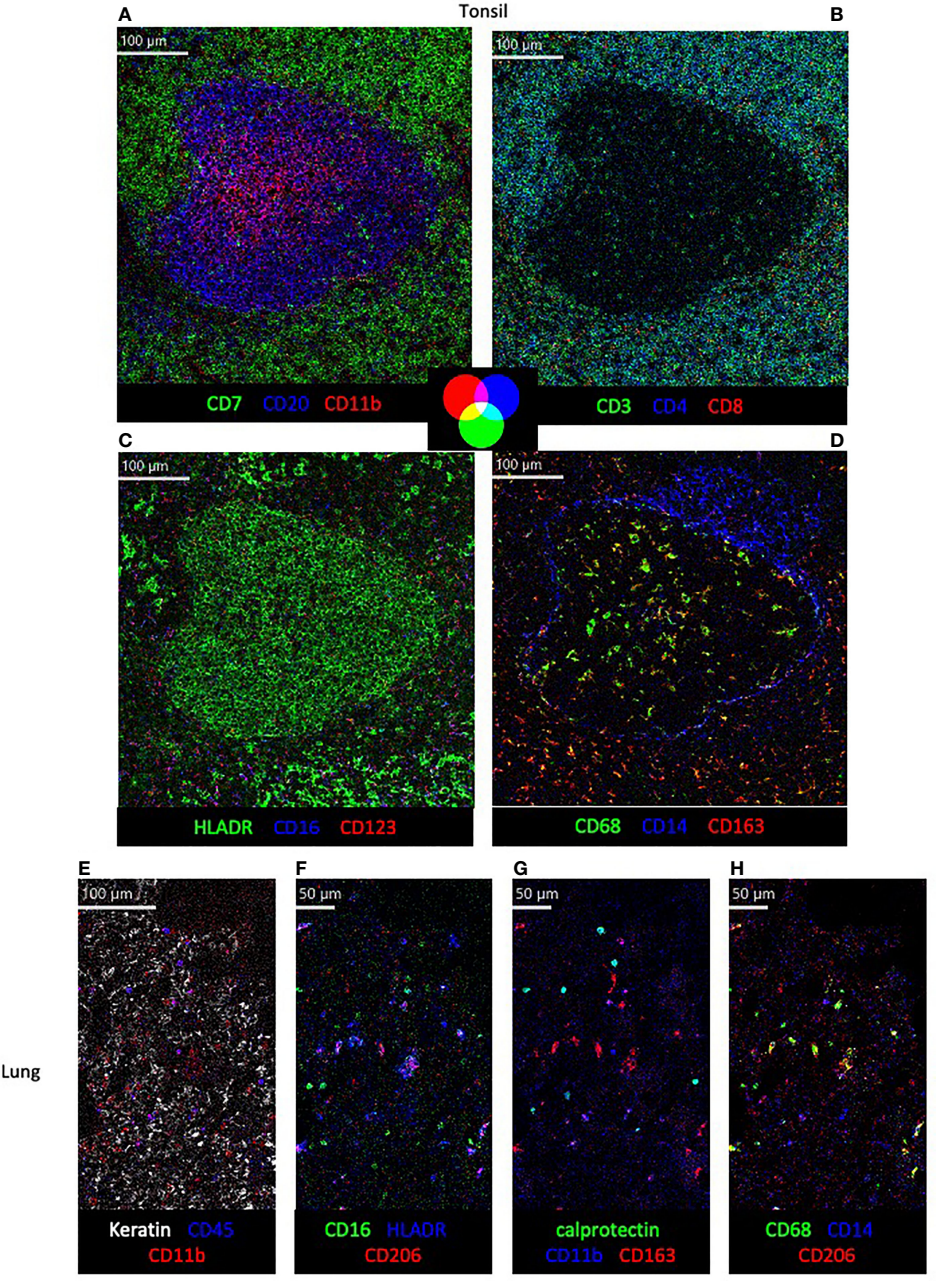
Visualization of structural markers and immune cells in non-human primate tissue by imaging mass cytometry. **(A–D)** in cynomolgus macaque tonsil tissue with color co-expression key. **(E–H)** Detection of structural **(E)** and immune subset markers **(E–H)** in rhesus macaque lung tissue.

To demonstrate the use of this panel in different tissues and primate species, tonsil and lung sections of rhesus and cynomolgus macaques were stained with the full panel of antibodies according to the conditions in **Table 1** (**Figure 3**). Multiple images showing expression of different combinations of three of the 18 markers on the same region of interest are used to assess co-expression of relevant markers. Visualization of all 18 markers, and their respective co-expression patterns is not possible at once due to limited color palette that can be discriminated by the eye, but in future will be explored with high-dimensional analysis tools. While the initial testing of antibody reactivity was performed in rhesus macaque tissue, all antibodies also performed in cynomolgus tissue in full panel staining, as was expected due to the species close relationship. In the tonsil, organization of CD20^+^ B cell, CD7^+^ T cells/ILCs and CD11b^+^ myeloid cells in and around a follicle is shown (**Figure 3A**), followed by a more detailed representation of CD4^+^ and CD8^+^ T cell phenotype (**Figure 3B**). In addition, the mutually exclusive expression of myeloid markers HLADR, CD16, CD123 and CD14 are shown, as well as the co-expression of CD163 and CD68 outside of the follicle (**Figures 3C, D**). In the lung, the first panel shows the distribution of keratin^+^ epithelial cells, CD45^+^ leukocytes and CD11b^+^ myeloid cells (**Figure 3E**). The other panels demonstrate myeloid marker expression patterns in the lung, showing co-expression of HLADR with CD206, calprotectin with CD11b and CD68 with CD206, but not between CD16 and CD206, CD11b and CD163 or CD14 and CD68 (**Figures 3F–H**).

## Discussion

Here, we describe a pipeline to test suitability of antibodies for FFPE NHP tissue and its application in the development of an 18-marker immunophenotyping panel for IMC on NHP tissue.

In animal studies, tissue is often preserved in FFPE blocks which has resulted in large archives of material representing a wide range of conditions in communicable and non-communicable diseases. Until recently, such blocks were mainly used for IHC analyses with the capacity to identify one, or when using consecutive slides a few, major cell subset(s) such as CD4^+^ or CD8^+^ T cells and B cells. As our knowledge of immunology expands, it is clear that simultaneous detection of more markers is needed to identify functionally distinct subsets within these general populations. Novel techniques such as IMC make this possible. The main challenge for its application on NHP FFPE tissue is the identification of suitable reagents, as NHP reagents are mainly validated for use in flow cytometry or frozen tissue IHC. As the number of targets with FFPE-validated anti-NHP antibodies is limited, we mainly used much more abundantly available FFPE-approved anti-human antibodies. In order to efficiently select suitable clones, we adapted an existing approach of IMC panel development to create a pipeline to test cross-reactivity of anti-human antibodies in NHP tissue (10).

The inclusion of human tissue in the first step of the pipeline enables rapid assessment of appropriate staining pattern in the NHP tissue by comparison. Eight out of thirteen antibodies that were deemed unsuitable for use in IMC were lost in this first step, meaning that the inclusion of this step enables quick exclusion of the majority of non-cross-reactive antibody and thereby limits loss of time and material.

All but one of the antibodies were conjugated in-house as the number of clones available in metal-conjugated format is very limited and even fewer are cross-reactive in NHP tissue. Furthermore, as the current panel was designed as a backbone to customize with markers of interest for specific research questions, self-conjugating gave us additional flexibility to place the lineage markers on dimmer metal isotopes and leave the brighter available for potentially dimmer targets. By performing conjugations in-house the full breadth of the isotopes available can be used for expansion of the panel.

As the panel presented here consists of anti-human antibodies, findings in NHP tissue can be verified in relevant human material using the same panel (**Supplementary Figure 1**). Furthermore, as banking of study material in FFPE format is standard for labs globally and transportation of such material has no specific requirements, IMC can be a valuable tool to compare tissues from similar NHP studies performed in different labs or outcomes from recent and historic studies.

Recent awareness of the importance of spatial context for cellular function underlines the need for technologies that can simultaneously gather information on tissue organization and detailed cellular phenotype. IMC is such a technology and can readily be applied on archived FFPE material. NHP have been an important tool in studying the immunology behind disease and vaccination for many years. The pipeline described here provides a step-by-step approach to select and incorporate antibodies in an IMC panel suitable for NHP tissue by verification of cross-reactivity by direct comparison with staining in human tissue. The panel presented identifies the main leukocyte lineages and subsets, with the possibility to add extra markers of interest. By sharing this pipeline and panel, we hope to contribute to maximizing the output of (already performed) NHP studies and ultimately increased insight into spatial organization of cells within tissues and their role in protection or susceptibility to disease.

## Data availability statement

The raw data supporting the conclusions of this article will be made available by the authors, without undue reservation.

## Ethics statement

NHP samples were obtained from the Biomedical Primate Research Centre (BPRC), Rijswijk, the Netherlands (courtesy of DVM I. Kondova). BPRC is licensed by the Dutch authority to breed non-human primates and to use them for research in areas of life-threatening and disabling diseases without suitable alternatives. BPRC complies to all relevant legislation with regard to the use of animals in research; the Dutch ‘Wet op de Dierproeven’ and the European guideline 2010/63/EU. BPRC is AAALAC accredited since 2012. Tissue samples from purpose-bred rhesus and cynomolgus macaques in the present study, were retrieved from a biobank of materials that become available occasionally, when an animal happens to be indicated for ketamine-sedation and euthanasia for veterinary and animal welfare reason. Thus, the availability of samples was exploited as it occurred, beyond any legal requirement for prior approval of protocol as there was no pre-existing study plan nor any discomfort afflicted to animals for the sake of a research objective.

## Author contributions

PN performed the experiments and wrote the manuscript. MI provided IMC expertise, provided human FFPE blocks and evaluated titration results. FV provided NHP material. TO supervised the study and revised the manuscript. SJ initiated and supervised the study, and revised the manuscript. All authors contributed to the article and approved the submitted version.

## Conflict of interest

The authors declare that the research was conducted in the absence of any commercial or financial relationships that could be construed as a potential conflict of interest.

## Publisher’s note

All claims expressed in this article are solely those of the authors and do not necessarily represent those of their affiliated organizations, or those of the publisher, the editors and the reviewers. Any product that may be evaluated in this article, or claim that may be made by its manufacturer, is not guaranteed or endorsed by the publisher.

## Supplementary material

Supplementary Table 1 Antibodies not included in the imaging mass cytometry panel and reason for exclusion.
