# Supplementary material for: An imaging mass cytometry immunophenotyping panel for non-human primate tissues
Source: Front Immunol. 2022 Jul 15;13:915157. doi: 10.3389/fimmu.2022.915157 (PMC9334813; doi:10.3389/fimmu.2022.915157)
Supplement: Supplementary Table 1 — Antibodies not included in the imaging mass cytometry panel and reason for exclusion. [file Table_1.docx]

Supplementary table 1. Antibodies not included in the imaging mass cytometry panel and reason for exclusion

| Target | Clone | Supplier | Working in NHP | pH antigen retrival | Reason for exclusion |
| --- | --- | --- | --- | --- | --- |
| CD1c | EPR23189-196 | Abcam | no | - | not suitable for NHP |
| CD8 | C8/144B | CST | no | - | not suitable for NHP |
| CD11c | EP1347Y | Abcam | no | - | not suitable for NHP |
| CD15 | BRA-4F1 | Abcam | no | - | not suitable for NHP |
| CD19 | EPR5906 | Abcam | yes | pH 6 | not relevant for this panel |
| CD45 | D058-1283 | BD | no | - | not suitable for FFPE |
| CD66abce | TET2 | Abcam | no | - | not suitable for NHP |
| CD103 | SP301 | Abcam | yes | pH 6, pH 9 | not relevant for this panel |
| CD117 | D3W6Y | CST | yes | pH 6, pH 9 | no signal on IMC |
| CD161 | OTI1D8 | Abcam | no | - | not suitable for NHP |
| CD161 | 14F1F11 | Novus | no | - | not suitable for NHP |
| CD163 | EPR14643-36 | Abcam | no | - | not suitable for NHP |
| CD163 | EPR19518 | Abcam | yes | pH 6, pH 9 | no signal on IMC |
| EPCAM | EPR20532-222 | Abcam | yes | pH 6 | not relevant for this panel |
| γδTCR | B1 | Biolegend | no | - | not suitable for FFPE |
| NKG2A/C | Z199 | Beckman Coulter | no | - | not suitable for FFPE |
