# Supplementary figures and images for: An imaging mass cytometry immunophenotyping panel for non-human primate tissues
Source: Front Immunol. 2022 Jul 15;13:915157. doi: 10.3389/fimmu.2022.915157 (PMC9334813; doi:10.3389/fimmu.2022.915157)

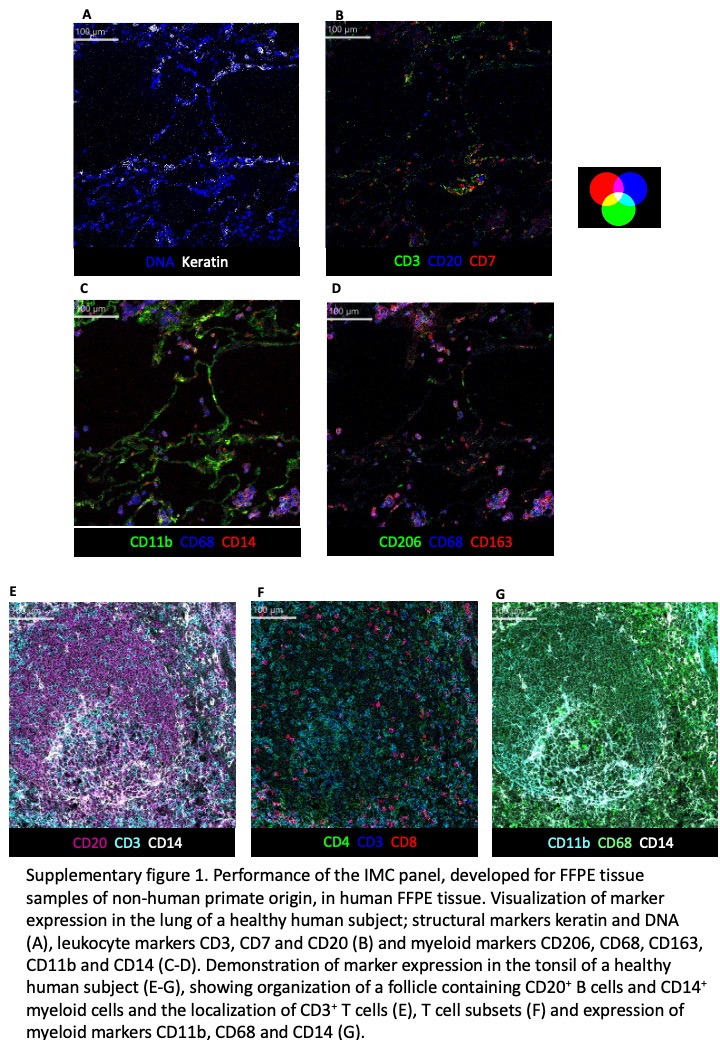

Supplement: Supplementary file 2 [file Image_1.jpeg]
